# Impact of Omega-3 Fatty Acids on the Gut Microbiota

**DOI:** 10.3390/ijms18122645

**Published:** 2017-12-07

**Authors:** Lara Costantini, Romina Molinari, Barbara Farinon, Nicolò Merendino

**Affiliations:** Department of Ecological and Biological Sciences (DEB), Tuscia University, Largo dell’Università snc, 01100 Viterbo, Italy; lara.cost@libero.it (L.C.); rominamolinari@libero.it (R.M.); barbara.farinon@gmail.com (B.F.)

**Keywords:** omega-3 PUFAs, DHA, EPA, gut microbiota, dysbiosis, inflammation, behavioral disorders

## Abstract

Long-term dietary habits play a crucial role in creating a host-specific gut microbiota community in humans. Despite the many publications about the effects of carbohydrates (prebiotic fibers), the impact of dietary fats, such as omega-3 polyunsaturated fatty acids (PUFAs), on the gut microbiota is less well defined. The few studies completed in adults showed some common changes in the gut microbiota after omega-3 PUFA supplementation. In particular, a decrease in *Faecalibacterium*, often associated with an increase in the *Bacteroidetes* and butyrate-producing bacteria belonging to the *Lachnospiraceae* family, has been observed. Coincidentally, a dysbiosis of these taxa is found in patients with inflammatory bowel disease. Omega-3 PUFAs can exert a positive action by reverting the microbiota composition in these diseases, and increase the production of anti-inflammatory compounds, like short-chain fatty acids. In addition, accumulating evidence in animal model studies indicates that the interplay between gut microbiota, omega-3 fatty acids, and immunity helps to maintain the intestinal wall integrity and interacts with host immune cells. Finally, human and animal studies have highlighted the ability of omega-3 PUFAs to influence the gut–brain axis, acting through gut microbiota composition. From these findings, the importance of the omega-3 connection to the microbiota emerges, encouraging further studies.

## 1. Introduction

In the last few years, the emergence and growing accessibility of next-generation sequencing (NGS) technologies have allowed advances in the understanding of the composition and functional activity of the gut microbial community. Approximately 100 papers on gut microbiota were published in 2007, whereas about 3000 such studies were published in 2016, and almost the same number to date in 2017 (research performed by setting the words “gut” and “microbiota” in October 2017 on PubMed and Scopus). The importance of using NGS technology is due to the necessity of simultaneous analysis of a large amount of genetic material. Indeed, overall, the gut microbiota is estimated to contain 150 times more genes than the human genome. These genes have been estimated to belong to approximately 10^13^–10^14^ microbes, with a species diversity of up to several hundred per individual [1]. However, The Human Microbiota Project and other studies have collectively found that thousands of different species may inhabit the human gut, pointing out the high degree of taxa variation in the microbiota composition of different populations. Despite this variation, the human gut microbiota is characterized by some basic similarities. Approximately 60% of the gut bacteria belong to the *Bacteroidetes* and *Firmicutes* phyla, and, among them, *Bifidobacterium*, *Lactobacillus*, *Bacteroides*, *Clostridium*, *Escherichia*, *Streptococcus*, and *Ruminococcus* are the most commonly found genera in adults [2]. However, several factors influence the bacterial composition in taxa type and abundance, making the total gut microbiota profile host-specific in humans. These factors include host phenotype, such as age, gender, body mass index (BMI), lifestyle, and immune function; geographical belonging and environmental factors; use of antibiotics, drugs, and probiotics; and diet. 

The causal relationship between the gut microbiota and overall pathological conditions is still unclear. Indeed, it is still unclear whether a disease-prone microbial composition exists (so-called dysbiosis) or whether the changes in the microbial community occur after the onset of the disease [3]. Conversely, diet undoubtedly influences the composition of gut microbiota, providing nutrients for both the host and the bacteria. This gut community has many degrading enzymes and metabolic capabilities that are able to break down macromolecules into smaller chemical compounds, which can then be uptaken by enterocytes [4]. Moreover, long-term dietary habits have been shown to play a crucial role in creating an inter-individual variation in microbiota composition [5]. However, despite the great number of publications on the effects of carbohydrates, the impacts of dietary fats and protein on the gut microbiota are less well defined. In particular, gut microbiota changes associated with omega-3 fatty acids are poorly understood.

Among the omega-3 polyunsaturated fatty acids (PUFAs), eicosapentaenoic acid (EPA, C20:5) and docosahexaenoic acid (DHA, C22:6) are the two main bioactive forms in humans. These fatty acids can be synthesized from the dietary precursor and essential fatty acid, α-linolenic acid (ALA, C18:3). However, the synthesis pathway requires several elongation and desaturation chemical reactions, so that the conversion of the two active forms in mammals is less efficient than dietary uptake. For this reason, consumption of EPA- and DHA-rich foods is recommended. However, since foods rich in these fatty acids are not widespread, EPA and DHA are widely used as nutritional supplements, often as nutraceuticals. Several papers have demonstrated the correlation between omega-3 PUFAs and the inflammatory response. Although the literature on this topic is discordant, omega-3 PUFAs are generally associated with anti-inflammatory effects, in comparison with the omega-6 PUFAs that are linked to pro-inflammatory effects, due to the different downstream lipid metabolites [6]. Also, with regards to the link to immunity, studies have shown that the supplementation of omega-3 PUFAs provides multiple health benefits against different chronic degenerative diseases, such as cardiovascular diseases [7], rheumatoid arthritis [8], inflammatory bowel disease (IBD) [9], depression [10], and cancer [11].

Considering the few insights existing in literature, in the present review, we assessed whether omega-3 PUFAs have an impact on the composition of the human gut microbiota in adults and infants. Moreover, a connection of this topic to inflammation and behavioral disorders was completed.

## 2. Omega-3 Influence on Human Gut Microbiota: State of the Art

The use of NGS technology has expanded the knowledge about the correlation between the human gut microbiota and omega-3 PUFAs. However, the literature in this topic is still in the initial stages. The current literature is listed below and summarized in Table 1. The first report in the literature about the impact of omega-3 fatty acids on human gut microbiota of adults came from a clinical study carried out in 60 overweight (BMI > 25) healthy people, between 40 and 60 years old. In this study, a commercially available probiotic with high concentrations of *Bifidobacteria*, *Lactobacilli*, and *Streptococcus thermophilus* (named VSL#3) was provided in combination with and without an omega-3 nutraceutical supplementation of 180 mg EPA and 120 mg of DHA for six weeks. This study failed to elucidate differences between the probiotic group and the probiotic plus omega-3 group. However, the limitation of this analysis was that the evaluation of microbiota changes was only completed using colony counting on anaerobic or aerobic selective media [12]. Subsequent studies focused more on food and diet impact instead of nutraceutical use of omega-3 PUFAs, likely because omega-3 fatty acids integrated in a food matrix can have a higher positive impact on gut microbiota. Supporting this hypothesis, a randomized crossover trial was completed on 20 middle-aged healthy individuals by administering a high daily dose (4 g) of a mixed DHA/EPA supplement for eight weeks [13]. The supplementation was performed using two different formulations: as a nutraceutical in the form of capsules, and as functional drink that was EPA- and DHA-rich. In this study, a taxonomy classification of the whole microbiota of the samples was completed with NGS technology. In this case, no statistically significant changes were observed in the *Firmicutes*/*Bacteroidetes* phyla ratio for both types of supplementations. On the contrary, analyzing the data at family and genus levels revealed consistent differences associated with both omega-3 PUFA supplementations. In particular, increases in the *Clostridiaceae*, *Sutterellaceae*, and *Akkermansiaceae* families were recorded, and these changes were reverted by the washout period. A statistically increased abundance of *Bifidobacterium* and *Oscillospira* genera, associated with a reduction of *Coprococcus* and *Faecalibacterium*, were found after both omega-3 PUFA supplementations in comparison with before the study and after washout. Instead, an increase in *Lachnospira* and *Roseburia* genera was prominent only after the functional omega-3 drink feeding. So, as previously anticipated, the functional drink had a greater impact on gut microbiota in comparison with nutraceutical supplementation. This study highlighted the increased abundance of butyrate-producing bacterial genera after omega-3 PUFA supplementation [13]. Acetate, propionate, and butyrate are the most abundant (>95%) short-chain fatty acids (SCFA) present in gut lumen, as end products of the fermentation of dietary fibers by the gut microbiota. Among the dominant butyrate-producing bacterial taxa, the following genera belonging to the *Lachnospiraceae* family of the phylum *Firmicutes* were found: *Eubacterium*, *Roseburia*, *Anaerostipes*, and *Coprococcus* [14]. The importance of butyrate, and SCFAs in general, are linked to anti-inflammatory properties. Indeed, they have been shown to ameliorate IBD, although their exact mechanism of action is still not completely clear [15].

In another analysis named COMIT (Canola Oil Multicenter Intervention Trial), a double-blinded randomized crossover clinical study, the effect of five different unsaturated oil blends on gut microbiota were tested in 25 volunteers with a risk of metabolic syndrome [16]. These participants were recruited based on the presence of at least one of these risk factors: wide waist circumference, high blood pressure, high triglyceride level, low HDL-cholesterol, and high blood glucose. The dietary treatment consisted of a daily intake of 60 g of one of the following dietary oils for 30 days: conventional canola oil (35.17 g oleic acid/60 g oil), DHA-enriched high oleic canola oil (37.95 g oleic acid and 3.48 g DHA/60 g oil), high oleic canola oil (42.88 g oleic acid/60 g oil), a blend of 25:75 corn/safflower oil (41.61 g linolenic acid/60 g oil), and a blend of 60:40 flax/safflower (22.48 g linolenic acid and 19.19 g ALA/60 g oil). After a pyrosequencing analysis, these dietary treatments revealed differences at the genus level rather than the phylum level. The high oleic canola oil feeding resulted in the highest level of *Faecalibacterium* among all other oils. Conversely, DHA-enriched high oleic canola oil resulted in the lowest level. A comparison between canola and canola/DHA indicated that canola was associated with *Coprobacillus* and *Blautia*, whereas canola/DHA was associated with the family *Lachnospiraceae* of the phylum *Firmicutes*. Instead, the comparison between all the canola oils and the PUFA-rich oils (i.e., corn/safflower and flax/safflower) revealed a correlation of the genera *Parabacteroidetes*, *Prevotella*, *Turicibacter,* and *Enterobacteriaceae* family with the first group versus the genus *Isobaculum*, associated with the second group. For the microbiota changes between canola and canola/DHA oils, the authors speculated that this could be the result of an interaction between the gut microbiota and DHA metabolites, potentially through the enterohepatic circulation of bile salts [16,17].

Another dietary intervention was the Pilchardus Study, a multicentre randomized trial in patients diagnosed with type 2 diabetes (glycated haemoglobin level between 6.0% and 8.0%) and not subjected to insulin treatment or antidiabetic drugs [18]. In this study, the participants followed a six-month dietary intervention of either a standard diet for diabetes, control (*n* = 15), or a standard diet supplemented with 100 g of sardines five days a week (*n* = 17), which provided approximately 3 g of EPA and DHA. The analysis of the abundance of the target bacteria by quantitative real-time polymerase chain reaction (qPCR) revealed a significant decrease in *Firmicutes* phylum in both experimental groups, with the *Firmicutes*/*Bacteroidetes* ratio decreasing in the omega-3 group. Moreover, *E. coli* concentrations increased in both groups and the proportions of *Bacteroides-Prevotella* increased in the sardine-fed group [18].

In another case report, Noriega and co-workers analyzed the effect of omega-3 PUFA supplementation on human gut microbiota using NGS technology [19]. In this study, a daily supplementation of 600 mg of omega-3 PUFAs through a fish protein diet was implemented for two weeks in one 45-year-old man. This intervention led to an increase in the *Firmicutes* phylum, and to a simultaneous decrease in *Bacteroidetes* and *Actinobacteria*. Moreover, a reduction in *Faecalibacterium* genus versus an increase in *Blautia*, *Roseburia*, *Coprococcus*, *Ruminococcus*, and *Subdoligranulum* genera was recorded. Some of these recorded genera are still associated with butyrate production. However, after two washout weeks, a reversal trend was observed, indicating that gut microbiota is strongly sensitive to diet changes [19].

The recent study of Menni and co-workers [20] correlated DHA circulating levels with DHA dietary intake, determined by a Food Frequency Questionnaire. The association with major taxa was determined in the largest population studied to date in this topic, with 876 participants, based on a cohort of middle-aged and elderly women (mean age = 64.98 years old). They found that a DHA intake of 350 mg/day resulted in a serum DHA concentration of 0.14 mmol/L, and was significantly associated with 36 Operational Taxonomic Units (OTUs). Of these, 21 OTUs (58%) belonged to *Lachnospiraceae*, 7 to *Ruminococcaceae* (19%), and 5 to *Bacteroidetes* (14%). In this study, a correlation between serum DHA and faecal metabolites was evaluated, and a positive correlation with *N*-carbamylglutamate was found. Even in this analysis, a positive correlation between omega-3 PUFAs and SCFA-producing bacteria (*Lachnospiraceae* family) was highlighted. The authors hypothesized that the levels of *N*-carbamylglutamate present in the gut lumen may mediate the association between the found taxa and serum DHA [20].

These studies have highlighted some common changes in gut microbiota after omega-3 supplementation. In particular, a decrease in *Faecalibacterium*, often associated with an increase in the *Lachnospiraceae* family, genus *Roseburia*, and *Bacteroidetes*, has been observed. In a cross-sectional study, the gut microbiota composition of IBD-affected individuals was identified [21]. Notably, in the IBD group, the authors found an increase in *Escherichia*, *Faecalibacterium*, *Streptococcus*, *Sutterella*, and *Veillonella* genera, whereas *Bacteroides*, *Flavobacterium*, and *Oscillospira* genera decreased [21]. Therefore, omega-3 PUFAs could improve IBD patients’ condition by reverting the microbiota to a healthier composition. Moreover, omega-3 PUFAs can trigger a healthy chain reaction, increasing SCFA amounts; their anti-inflammatory action can help improve this pathology. However, further studies are needed to validate this hypothesis.

Other studies focused on the correlation between gut microbiota changes and omega-3 diet in infants. Emerging evidence has shown that the acquisition of the microbiota community in infancy does not start from delivery, as long-believed, through natural parturition and subsequent breastfeeding, but rather begins in utero, demonstrated by the presence of a microbiota community in the placenta and amniotic fluid [22]. Therefore, the mother’s diet can influence the correct development of the infant’s microbiota during gestation.

The first evidence of the correlation between infant microbiota and omega-3 PUFAs came from the randomized, non-blinded, 2 × 2 intervention study by Nielsen and colleagues [23]. In this study, 114 nine-month-old infants were included and randomized to receive cow’s milk or infant formula with or without 5 mL/day of fish oil until the 12th month. In 65 of the 114 infants, the gut microbiota were analyzed in faeces by fingerprint profiles generated by the V3 and V6-8 PCR-DGGE (Denaturing Gradient Gel Electrophoresis). The study revealed that consumption of fish oil in cow’s milk groups created a differential fingerprint profile, and this difference was not found in the infant formula groups. The authors explained that difference with the fact that cow’s milk contains considerably less omega-3 PUFAs in comparison with infant formula, so omega-3 PUFAs can have a dose-response effect in changing the gut microbiota profile [23]. Subsequently, the same research group performed a double-blinded randomized parallel intervention in 132 nine-month-old infants, to analyze microbiota differences after nine months of daily supplementation with 5 mL fish oil (1.6 g EPA and DHA) or sunflower oil (3.1 g linolenic acid, C18:2 omega-6) [24]. Differences between groups were analyzed in faeces using fingerprint profiles generated analyzing Terminal Restriction Fragment Length Polymorphism (T-RFLP). Interestingly, the authors found that fish oil caused significant changes in the microbiota in comparison with sunflower oil, but only among children who had stopped breastfeeding before the study. The authors determined that the cessation of breastfeeding opened the infant microbiota to new bacteria. Therefore, breastfeeding likely causes a delay in gut microbial maturation. Indeed, they found that the T-RFLP pattern of non-breastfed infants at the 9th month was more similar to those at the 18th month than that of the partial breastfed nine-month-old infants [24].

However, the first deeper analysis in infants using NGS technology was a randomized controlled trial, where 32 infants born premature with enterostomy were randomized to receive either the usual nutritional therapy or an enteral supplementation of a fish and safflower blend oil until bowel reanastomosis, for a maximum of 10 weeks [25]. The experimental PUFA group showed greater bacterial diversity combined with lower abundance of some pathogenic bacteria, such as *Streptococcus*, *Clostridium*, and some genera of the *Enterobacteriaceae* family, such as *Escherichia*, *Pantoea*, *Serratia*, and *Citrobacter* [25]. In a population-based prospective human cohort study [26], 81 maternal-neonate dyads were studied to understand whether a maternal high-fat diet can influence the neonatal and infant gut microbiota. Stool and meconium were collected from neonates until six weeks of age, and a dietary questionnaire was completed by the mothers to estimate fat, sugar, and fiber intakes. From the questionnaire, two different groups were identified: a high-fat maternal diet group, with a 43.1% fat intake, above the recommended limit of 20–35%, and a low-fat maternal diet group, with a 24.4% fat intake. This cohort analysis revealed that a maternal high-fat diet during gestation influenced the neonatal microbiota, resulting in a significant depletion of *Bacteroides* in the high-fat maternal diet group that persists beyond delivery, in infants four to six weeks old [26]. In that study, fatty acid types were not differentiated. However, considering that the levels for sugar and fiber intakes were not in line with the recommended range (i.e., sugar mean 59.6%, recommended <25%; fiber mean 24.9%, recommended >25%), the main fat intake was assumed to be from saturated fatty acids, common in the Western American diet. Therefore, as discussed above, the omega-3 PUFAs favor the butyrate-producing bacterial genera, whereas a diet rich in saturated fats can depauperate the gut microbiota of these commensal bacteria.

## 3. Gut Microbiota; Inflammation; and Omega-3

Several studies have shown that the intestinal microbiota is important for the development of the systemic and gut immune response [27,28]. Studies on germ-free mice have shown that the lack of intestinal microbiota leads to the reduced development of the intestinal immune system and oral tolerance [29]. Another role for the gut microbiota is the continuous stimulation of resident macrophages to release large amounts of IL-10 that promote the induction of regulatory T cells (Treg) and prevent excessive development of T helper 17 (Th17) cells [30]. Symbiotic intestinal bacteria are essential for the development and function of specific lymphocyte subsets. Early exposure to microbes in the intestine could be a critical factor modulating the original Th2-biased immune response, to subsequently induce the differentiation of other Th cell lineages, such as Th1, Th17, and Treg cells [31].

The gut microbiota produces many immunogenicity endotoxins such as lipopolysaccharides (LPS). In some cases, LPS pass through the intestinal wall, especially when the barrier is destroyed, causing further damage. Even minute quantities of LPS in the systemic circulation, on the picogram scale, have the potential to elicit an inflammatory response in humans. LPS is thought to enter the circulation by transportation across the intestinal epithelium either via the para-cellular pathway through the openings of intestinal tight-junctions between two epithelial cells, or through a trans-cellular pathway [32].

Inflammation plays a role in the insurgence of various diseases and recent findings have suggested that an altered gut microbiota, in particular a reduction of health-promoting gut bacteria such as *Lactobacilli* and *Bifidobacteria*, has been linked to metabolic diseases, including obesity, diabetes, cardiovascular diseases [33], cystic fibrosis [34], neurological diseases (Parkinson’s disease, Alzheimer’s disease, and multiple sclerosis) [35], as well as musculoskeletal conditions such as frailty, osteoporosis, and gout [36,37].

As mentioned above, diet is one of the strongest selective pressures for microbial communities within the gastrointestinal tract. Table 2 summarizes the studies that have investigated the role of PUFAs on microbiota. Several studies have demonstrated that feeding a high-fat diet (i.e., 45–60% kcal from fat) influences the types and amounts of gut microbes and adversely affects intestinal health. In particular, a high-fat diet is implicated in dysbiosis, including a decrease in *Bacteroidetes* and an increase in both *Firmicutes* and *Proteobacteria* in the murine model [38,39], a reduction of microbiota richness in terms of the number of species per sample [40,41], as well as an increase in LPS-producing bacteria such as *Enterobactericeae* and/or a decrease in LPS-suppressing bacteria (those which can lower the numbers of LPS-producing bacteria, such as *Bifidobacterium*). Moreover, a high-fat diet results in epithelial alterations, such as intestinal barrier dysfunction [42]; a higher intestinal permeability [43,44]; and an increased LPS translocation that can diffuse from the gut to the bloodstream, either by direct diffusion mediated by para-cellular permeability or through absorption by enterocytes during chylomicron secretion [45]. Current evidence suggests that dietary fat augments the circulating LPS concentrations. The resultant postprandial endotoxemia leads to low-grade systemic inflammation, which has been implicated in the development of several metabolic diseases, insulin resistance, adipocyte hyperplasia and reduction of pancreatic β-cell function [46], and impaired glucose metabolism [47].

Studies have shown that different types of dietary fat, including saturated fatty acids (SFAs), monounsaturated fatty acids (MUFAs), and PUFAs, and their abundance in the diet, could change gut microbiota composition [48]. In particular, omega-3 PUFAs share the important immune system activation/inhibition pathway with gut microbes modulating pro-inflammatory profiles [49]. For example, supplementation with an equal mixture of EPA and DHA decreased intestinal barrier dysfunction and decreased PPAR-γ levels caused by ischemia and reperfusion intestinal injury in a Sprague Dawley rat model [50]. Several types of fatty acids have an antimicrobial activity, and this activity occurs after the complete enzymatic hydrolysis of fat by the gut microbiota in the lower gastrointestinal tract [51]. The antimicrobial activity of fatty acids depends on the length of their carbon chain and on the presence, number, position, and orientation of double bonds. Unsaturated fatty acids tend to have greater activity than saturated fatty acids with the same length carbon chain [51]. The antimicrobial activity of PUFAs increases in the direction of the number of double bonds in their carbon chain; the cis-orientation seems to have more activity than the trans-orientation. Some studies have shown that omega-3 PUFAs can modify the intestinal microbiota composition [52] by increasing the number of *Bifidobacteria* that decrease gut permeability [53], and increase the number of *Enterobacteria* that increase intestinal permeability [54], allowing increased systemic concentration of LPS and endotoxemia.

Studies on the effects of omega-3 PUFAs on microbiota have mainly focused on the major bacterial phyla *Bacteroidetes* and *Firmicutes* in animal models. Omega-3 PUFAs from flaxseed seem to decrease the proportion of *Bacteroidetes* [55], and those from fish oil appear to lower the population of *Firmicutes* [56]. An increase in the *Firmicutes*/*Bacteroidetes* ratio has been linked to weight gain and other metabolic conditions, such as insulin resistance, in part by the synthesis of SCFAs.

Caesar and colleagues [57] showed that the type of dietary fat is a major driver of community structure, affecting both the composition and diversity of the gut microbiota. The authors fed two different groups of rats either a fish-oil diet or a lard diet. The results showed that mice fed fish oil had higher levels of *Lactobacillus* and *Akkermansia muciniphila* than mice fed with lard, in which *Bilophila* was abundant. The increase of *Lactobacillus* is associated with reduced inflammation in several inflammatory bowel diseases. The increase of *Akkermansia muciniphila* improves the barrier function and glucose metabolism, and also decreases macrophage infiltration in the white adipose tissue (WAT) [58]. In a study comparing different types of high-fat diets on the profile of gut bacteria in a mouse model, Liu and co-workers [55] observed that consumption of an SFA-rich diet resulted in a significant decrease in the abundance of *Bacteroidetes* compared to either omega-3 PUFA-rich or omega-6 PUFA-rich diets. A mouse study [59] reported that a diet supplemented with EPA and DHA significantly increased the abundance of *Firmicutes* and reduced the percentage of *Bacteroidetes*, compared with a diet supplemented with oleic acid. As for human studies [16,17], the changes in metabolic parameters after DHA intake in mice could be the result of interactions between gut microbiota and DHA metabolites, potentially through the enterohepatic circulation of bile salts [17]. Myles et al. [60] indicated that omega-3 PUFA intake in pregnant mice could influence offspring microbiota and immune response through the anti-inflammatory effects of omega-3 PUFAs. These findings suggest that the administration of omega-3 PUFAs during embryonic development is important for the proper development of the microbiota and immune system.

Studies on mice-transplanted faeces showed that the omega-3 PUFAs can modify the microbiota through the production and secretion of intestinal alkaline phosphatase (IAP), leading to a reduction in the number of LPS-producing bacteria, thus reducing metabolic endotoxemia [52]. Mujico et al. [59] showed that, in diet-induced obese mice, supplementation with a combination of EPA and DHA significantly increased the quantities of *Firmicutes*, and especially the *Lactobacillus* taxa. Evidence suggests that some physiological effects of the microbiota could be associated with the interactions between dietary PUFAs. Dietary PUFAs have been suggested to affect the attachment sites for the gastrointestinal microbiota, possibly by modifying the fatty acid composition of the intestinal wall [61]. Data from animal models indicates that fish oil in particular has effects on shaping the microbiome. Ghosh et al. [62] found that mice fed a diet supplemented with fish oil had a reduced abundance of *Enterobacteriaceae* and *Clostridia* species compared with mice fed a diet rich in omega-6 fatty acids.

The role of omega-3 on microbiota composition and diversity has not yet been thoroughly explored in human cohorts in comparison to animal models. As described above, increased intestinal permeability is involved in several disorders associated with chronic low-grade inflammation, including obesity, obesity-associated insulin resistance, type 2 diabetes, and IBD. The integrity of the intestinal epithelium is created by the tight junctions. Tight junctions are composed of multiple proteins, including cytosolic zonula occludin. Zonulin, a detectable protein in human serum [63], has been shown to reflect intestinal permeability [64,65]. Serum zonulin has been used as a serum marker for intestinal permeability in several studies [66,67,68]. Increased serum concentrations have been detected in a range of metabolic conditions associated with chronic low-grade inflammation. This marker was used by Mokkala and co-workers [69] to analyze intestinal permeability in pregnant women. Numerous metabolic alterations accompany pregnancy that support foetal growth and development. Initial results suggested that healthy pregnant women exhibited an increase in intestinal permeability compared with non-pregnant women [70]. However, little is known about the effects of pregnancy on intestinal permeability and whether this could lead to subsequent health consequences.

Mokkala et al. [69] showed that gut microbiota composition, including both microbiota richness and the abundance of specific taxa, and dietary intakes of omega-3 PUFAs, fibers, and certain vitamins and minerals, are linked to concentrations of serum zonulin. The gut microbiota richness differed between the high and low zonulin groups, as exhibited by higher microbiota richness in the low zonulin group. Mokkala et al. [69] found that a higher total intake of omega-3 PUFAs was associated with lower serum zonulin concentrations. This was the first study to suggest that gut microbiota richness is associated with intestinal permeability in humans in vivo. This study on pregnant women showed a higher abundance of *F. prausnitzii* together with a lower abundance of *Bacteroides* in the low zonulin group, indicating that these bacteria may play a role in intestinal epithelial integrity. In a previous study [71], bacterial diversity was associated with intestinal barrier function in patients with ulcerative colitis. This observation may be important for human health because a high amount of pro-inflammatory species, such as *Bacteroides*, in relation to potentially anti-inflammatory species, such as *F. prausnitzii*, has been associated with adverse metabolic outcomes, such as insulin resistance. Instead, a higher abundance of the genus *Blautia* has been associated with glucose intolerance [72].

In maintaining intestinal epithelial integrity, PUFAs influence the inflammatory status of the gut by serving as precursors to anti-inflammatory eicosanoid synthesis, or enhance intestinal integrity by regulating the tight junction functions [73,74].

The pathology of IBDs, which include ulcerative colitis (UC) and Crohn’s disease (CD), is a chronic inflammatory condition of the gastrointestinal tract. Several studies have indicated that the intestinal microbiota is one of the critical factors influencing UC and CD [75]. Studies in patients with UC or CD showed an altered composition of gut microbiota with an increase in *Actinobacteria* and *Proteobacteria*, and a decrease in *Bacteroidetes* and *Firmicutes* [76,77].

In CD patients, Joossens et al. [78] observed a reduced concentration of *F. prausnitzii*, *B. adolescentis*, and *D. invisus*, and an increased abundance of *R. gnavus*. *F. prausnitzii* is a butyrate-producing bacterium; its decline leads to a decrease in SCFA production in IBD, whereas an increase was noted in sulfate-reducing bacteria that induce mucosal inflammation [79]. In IBD, the prolonged activation of NF-κB leads to the production of pro-inflammatory cytokines [80]. Omega-3 PUFAs inhibit the NF-κB pathway through resolvins and protectins. Based on the analyzed studies, omega-3 PUFAs may be a useful tool in the prevention of diseases associated with dysbiosis. Future studies with clinical trials are needed to analyze the relationship between omega-3 PUFAs and microbiota.

## 4. Gut Microbiota, Behavioral Disorders, and Omega-3

Inflammation and dysbiosis are conditions associated with different behavioral, mood, and psychological disorders, including major depressive disorder (MDD), anxiety, and autism spectrum disorder (ASD). Increasing evidence shows that gut microbiota influences mammalian behavior. For instance, the complete absence of microbiota in germ-free mice induced depressive-like behavior and impairments in sociability [81], whereas bacterial colonization of these mice improved social behavior [82]. Furthermore, psychological disorders, such as MDD and ASD, are characterized by higher intestinal permeability, chronic low-grade inflammation [83], neurotransmitter signaling alteration, and Hypothalamic–Pituitary–Adrenal (HPA) axis dysfunction [84], leading to excessive stress-induced corticosterone release. These are all processes that are influenced by gut microbiota. Indeed, the gut microbiota is an integral part of the microbiota–gut–brain axis, a bidirectional crosstalk between the gut microbiota and brain, essential in the regulation of many physiological functions, such as digestive and gastrointestinal functions, as well as inflammation, neurogenesis, neurodevelopment [85], behavior, and stress responses. Through this axis, the gut microbiota and central nervous system (CNS) communicate by different pathways, including endocrine, immune, and neural pathways, using the gastrointestinal tract as a scaffold [86]. 

Both MDD and ASD are characterized by similar alterations in gut microbiota composition associated with a pro-inflammatory microbial profile [81,87,88]. Since gut microbiota can modulate neurogenesis, neurodevelopment, and mammalian behavior, and since dysbiosis is linked to inflammation, neurodevelopmental, and behavioral disorders, correct microbiota development appears to be fundamental to guaranteeing proper brain function and avoiding behavioral and social impairments later in life. Various environmental factors that impair gut microbiota composition may impact neurodevelopment and increase the risk of behavioral disorders. With respect to this, omega-3 PUFAs, and in particular EPA and DHA, are essential nutrients for brain development and health as they play a pivotal role in the regulation of synaptic plasticity, neurogenesis [89], dopaminergic and serotonergic neurotransmission [90], and HPA axis activity [91]. An omega-3 PUFA deficiency, especially during intrauterine and early life, is associated with impaired psychomotor development, and issues with attention, cognition, and visual acuity [92]. Moreover, a substantial decrease in plasma and brain omega-3 PUFAs levels, for DHA in particular, was found in patients with ASD [93,94]; it is also correlated with mood and behavioral disorders such as anxiety and depression later in life [64,95]. Conversely, DHA supplementation has been shown to improve the symptoms of these conditions [96,97,98]. These omega-3 PUFA benefits on the brain may be due to their ability to modulate gut microbiota composition. 

To date, data are limited showing that omega-3 PUFA administration leads to benefits for behavioral disorders by modulating gut microbiota composition; the few studies on this subject, mostly completed in animal models, are summarized in Table 3. For instance, Pusceddu and colleagues [99] showed that long-term EPA/DHA administration can lead to a beneficial anti-inflammatory effect associated with a composition restoration of altered gut microbiota on maternal-separated rats. Particularly, maternal-separated rats showed an increase in *Bacteroidetes,* and non-separated rats showed a decrease in *Firmicutes*, in agreement with the results obtained by Jiang et al. [88] on depressed human patients. In these early-life-stressed rats, a long-term administration of EPA/DHA led to the restoration of the normal *Firmicutes/Bacteroidetes* ratio. Furthermore, long-term EPA/DHA administration in separated mice improved the inflammatory condition typically associated with stress by increasing the abundance of butyrate-producing bacteria and decreasing the levels of pro-inflammatory bacterial genera, such as *Akkermansia* and *Flexibacter,* which have been reported to be related to an inflammatory state [100,101]. These taxa changes align with those noted in a previously-mentioned case report [19]. Since inflammation plays an important role in depression, the gut microbiota shift observed in maternal-separated rats is likely protective of the behavioral disorders.

At a more molecular level, Kaliannan and co-workers [52] provided information about how omega-3 PUFAs modulate gut microbiota composition by enriching it with beneficial species through the modulation of IAP expression. Nevertheless, how omega-3 is able to modulate IAP expression must be clarified. One hypothesis is that lipid mediators obtained by omega-3 PUFA metabolizing, such as resolvin E1, are directly responsible for IAP expression [102].

Outcomes from another study by Davis et al. [103] on the stress-induced adult mouse model through social isolation demonstrated that environmental stress can cause significant changes in the adult gut microbiota, and these changes may be countered with the introduction of DHA into the diet, providing evidence of the ability of omega-3 PUFAs to positively modulate gut microbiota composition. In this survey, a sexual dimorphism was found in response to stress and to DHA treatment, with adult males being more sensitive than females. Gut microbiota changes appearing in males after isolation are linked with depressive-like behavior and showed a decrease in bacteria implicated in SCFA production, such as *Allobaculum*, and an increase in those involved in tryptophan metabolism, such as *Ruminococcus* species. Of note, this bacterial genus was also found in high levels in ASD children [104] and may lead to an increase in tryptophan biosynthesis that has been found to be higher in males. Enrichment of tryptophan biosynthesis supposedly leads to an increase in the concentration of quinolinic acid, a neuroactive compound able to cross the blood–brain barrier that has been correlated with anxiety behavior.

Another recent study on mice by Robertson and co-workers [92] highlighted that *in utero* and early life omega-3 PUFA intake, particularly EPA and DHA, regulates the gut microbiota development influencing bacterial abundance and types in adolescence and adulthood, and affects social and communicative behavior throughout one’s lifespan. In particular, mice born from mothers fed a diet lacking in omega-3 PUFAs and themselves fed the same diet displayed anxiety and depressive-like behavior, as well as a cognitive and sociability impairment, compared with those fed an omega-3 PUFA-enriched diet. These behavioral features were significantly more obvious in adulthood than in adolescence. Furthermore, mice groups lacking omega-3 PUFAs displayed a systemic inflammation activated by high LPS plasma levels and altered HPA axis activity, as well as an imbalance in the normal *Firmicutes/Bacteroidetes* ratio. However, mice fed an omega-3-enriched diet showed significantly enhanced cognition, and dampened HPA-axis activity and inflammation, as well as an improved intestinal epithelial integrity due to a higher abundance of the *Bifidobacteria* genus.

This evidence supports the idea of a novel mechanistic hypothesis by which omega-3 PUFAs exert their beneficial effects on health, brain functions, and behavior by influencing gut microbiota composition and, thus, gut–brain axis functionality.

## 5. Conclusions

**Figure 1:**
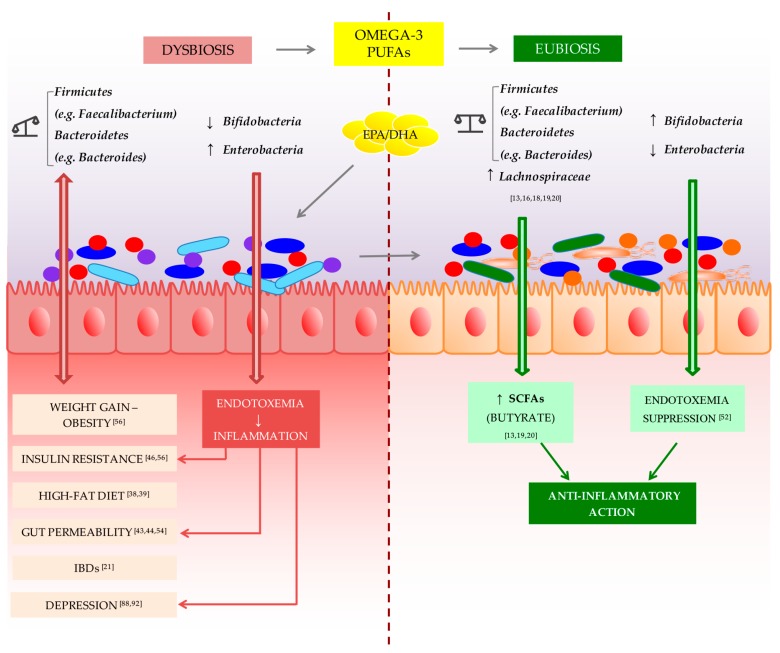
Omega-3 polyunsaturated fatty acid (PUFA) potential action in restoring eubiosis in gut microbiota. Dysbiosis of the *Firmicutes/Bacteroidetes* ratio is associated with several conditions, such as weight gain and obesity [56], insulin resistance [56], high-fat diet [38,39], gut permeability [54], IBDs [21], and depression [88]. Similarly, a *Bifidobacteria* decrease combined with a *Enterobacteria* increase leads to the establishment of endotoxemia that causes a chronic low-grade inflammation associated with some pathological conditions, like insulin resistance [46], gut permeability [43,44], and depression [92]. Initial evidence shows that omega-3 PUFAs are able to reverse this condition by restoring the *Firmicutes/Bacteroidetes* ratio, and increasing *Lachnospiraceae* taxa [13,16,18,19,20], both associated with an increased production of the anti-inflammatory short-chain fatty acid (SCFA) butyrate [13,19,20]. Moreover, animal studies showed the ability of omega-3 PUFAs to increase lipopolysaccharide (LPS)-suppressing bacteria, *Bifidobacteria*, and to decrease LPS-producing bacteria, *Enterobacteria*, negating the endotoxemia phenomenon [52]. For all these actions, omega-3 PUFAs can be considered as prebiotics, able to restore gut eubiosis in some pathological conditions.

The evidence is growing for a correlation between gut microbiota dysbiosis and pathological status. In particular, some metabolic disorders of the host are thought to be associated with an inflammation-related composition of the gut microbiota. Different bacterial taxa modulate immune functionality that can play pro- and anti-inflammatory roles, and, thus, the composition of the microbiota community determines, in part, the level of resistance to infection and susceptibility to inflammatory diseases. Omega-3 PUFAs exert significant effects on the intestinal environment; on mood and cognitive functioning, such as anxiety and depression; and modulating the gut microbiota composition (Figure 1). In summary, based on conducted studies, the omega-3 PUFAs can be considered prebiotics. Therefore, the consumption of an omega-3-rich diet has been thought to be beneficial for health, but the gut microbiota changes in humans associated with omega-3 PUFAs are poorly understood. Future research with well-conducted clinical trials is needed to analyze the relationships between omega-3 PUFAs and the gut microbiota.

## Figures and Tables

**Table 1:** Summarized studies investigating the omega-3 influence on human gut microbiota.

Human Studies	Studied Population	Diets	Method	Main Outcomes
Rajkumar et al. (2014) [12]	60 overweight healthy people	Commercial prebiotic, named VSL#3, vs. VSL#3 + 180 mg EPA and 120 mg of DHA for 6 weeks	Colony counting on anaerobic or aerobic selective media	No difference between groups.
Watson et al. (2017) [13]	20 middle-aged healthy individuals	4 g of mixed DHA/EPA supplement (as capsules and functional drink) for 8 weeks	Sequencing by NGS (Illumina) of *16S rRNA* gene, V4 region	No difference for *Firmicutes*/*Bacteroidetes* phyla ratio. Increases in the *Clostridiaceae*, *Sutterellaceae*, and *Akkermansiaceae* families in both experimental groups. Increased abundance of *Bifidobacterium*, *Oscillospira*, associated with a reduction of *Coprococcus* and *Faecalibacterium* genera in both experimental groups. Increased abundance of *Lachnospira* and *Roseburia* genera only in functional drink group.
Pu et al. (2016) COMIT study [16]	25 volunteers with risk of metabolic syndrome	60 g of five different unsaturated oil blends for 30 days: conventional canola oil (35.17 g oleic acid), DHA-enriched high oleic canola oil (37.95 g oleic acid and 3.48 g DHA), high oleic canola oil (42.88 g oleic acid), a blend of 25:75 corn/safflower oil (41.61 g linolenic acid), and a blend of 60:40 flax/safflower (22.48 g linolenic acid and 19.19 g ALA)	Sequencing by pyrosequencing of *16S rRNA* gene, V1–V3 regions	No difference between groups at phylum level. Highest level of *Faecalibacterium* genus in high oleic canola oil, and lowest in DHA-enriched high oleic canola oil. Conventional canola was correlated with *Coprobacillus* and *Blautia* genera, whereas canola/DHA was associated with the family *Lachnospiraceae* of the phylum *Firmicutes*. All the canola oils are correlated with *Parabacteroidetes*, *Prevotella*, and *Turicibacter* genera, and with *Enterobacteriaceae* family versus the PUFA-rich oils (i.e., corn/safflower and flax/safflower) correlated with the genus *Isobaculum.*
Balfego et al. (2016) Pilchardus Study [18]	32 patients diagnosed with type 2 diabetes	Standard diet for diabetes supplemented with 100 g of sardines 5 days a week for 6 months (*n* = 17) (~3 g of EPA + DHA)	qPCR on target bacterial indicators	*Firmicutes*/*Bacteroidetes* phyla ratio decrease, while *Prevotella* genus increase in the omega-3 group.
Noriega et al. (2016) [19]	One healthy 45-year-old man	Daily supplementation of 600 mg of omega-3 PUFAs by fish protein diet, for 2 weeks	Sequencing by NGS (Ion Torrent) of *16S rRNA* gene, V4 region	Increase of the phylum *Firmicutes* and a decrease of *Bacteroidetes* and *Actinobacteria* phyla. Reduction in *Faecalibacterium* genus versus an increase in *Blautia*, *Roseburia*, *Coprococcus*, *Ruminococcus* and *Subdoligranulum* genera.
Menni et al. (2017) [20]	Cohort of 876 middle-aged and elderly women	DHA intake of 350 mg/day with a serum concentration of 0.14 mmol/L. (DHA dietary intake determined by Food Frequency Questionnaire)	Sequencing by NGS (Illumina) of *16S rRNA* gene, V4 region	This intake is correlated with 21 OTUs belonging to *Lachnospiraceae* family, 7 OTUs to the *Ruminococcaceae* family, and 5 to the *Bacteroidetes* phylum.
Nielsen et al. (2007) [23]	One hundred and fourteen 9-month-old infants	Cow’s milk or infant formula with or without 5 mL/day of fish oil until the 12th month	Fingerprint profiles generated by PCR-DGGE of *16S rRNA* gene, V6-8 and V3 regions	Fish oil in cow’s milk groups has a differential fingerprint profile, and this difference was not found in infant formula groups.
Andersen et al. (2011) [24]	One hundred and thirty-two 9-month-old infants	Daily supplementation of 5 mL fish oil (1.6 g EPA + DHA) or sunflower oil (3.1 g linolenic acid, omega-6) for 9 months	Fingerprint profiles generated by T-RFLP of *16S rRNA* gene, whole gene	Fish oil gave significant changes in microbiota in comparison with sunflower oil, but only among children who had stopped breast-feeding before the study.
Younge et al. (2017) [25]	32 premature infants with enterostomy	Usual nutritional therapy and an enteral supplementation of a fish and safflower blend oil for a maximum of 10 weeks	Sequencing by NGS (Illumina) of *16S rRNA* gene, V4 region	Lower abundance of some pathogenic bacteria as *Streptococcus*, *Clostridium*, *Escherichia*, *Pantoea*, *Serratia*, and *Citrobacter* genera.

**Table 2:** Summarized studies investigating the omega-3 influence on animal and human gut microbiota.

Studies	Studied Population	Diets	Main Outcomes
Hildebrandt et al. (2009) [38]	C57BL/6 and β resistin-like molecule β knockout mice	High-fat diet (45% fat) for 21 weeks	High fat diet caused changed in microbiota composition with a decrease in *Bacteroidetes* phylum and an increase in both *Firmicutes* and *Proteobacteria* phyla.
Zhang et al. (2010) [40]	*Apoa-I−/−* and wild-type C57BL/6J mice	High-fat diet (34.9% fat) for 25 weeks	Sulphate-reducing, endotoxin-producing bacteria populations were enhanced in all animals fed with the high-fat diet.
Devkota et al. (2012) [41]	C57BL/6 germ free mice	Milk, lard fat, or PUFAs (38% fat) for 3 weeks	Milk fat promotes expansion of sulfite-reducing bacteria, *Bilophila* genus of *Proteobacteria* phylum. PUFAs resulted in a higher abundance of *Bacteroidetes* phylum and lower abundance of *Firmicutes* phylum.
Kaliannan et al. (2015) [52]	C57BL/6 wild type, fat-1 mice	Diet high in omega-6 PUFAs (10% corn oil) or omega-3 PUFAs (5% corn oil, 5% fish oil) for 8 months	High tissue omega-6/omega-3 PUFAs ratio can increase the proportions of LPS-producing and/or pro-inflammatory bacteria, low n-6/n-3 PUFAs ratio can increase LPS-suppressing and/or anti-inflammatory bacteria.
Liu et al. (2012) [55]	Wild-type mice	Saturated fatty acids, omega-6 PUFAs, or omega-3 PUFAs diet for 14 weeks	Omega-6 PUFAs and the omega-3 PUFAs diet reduced the proportion of *Bacteroidetes* phylum.
Yu et al. (2014) [56]	Imprinting Control Region mice	Natural saline group, high-dose fish oil group (10 mg/kg), and low dose fish oil group (5 mg/kg) for 2 weeks	Fish oil treatment resulted in a decrease in *Firmicutes* phylum.
Caesar et al. (2015) [57]	C57Bl/6 Wild-type germ free mice	High fat diet (45%) for fish oil or lard	Fish-oil diet increases levels of *Lactobacillus* genera and *Akkermansia muciniphila* species, lard diet increases levels of *Bilophila* genus of *Proteobacteria* phylum.
Mujico et al. (2013) [59]	Imprinting Control Region mice	Control diet (4% fat), high fat diet (43.3% fat, saturated 16.1%, MUFAs 12.7%, PUFAs 5.5%) for 19 weeks	PUFAs increases *Firmicutes* phylum.
Ghosh et al. (2013) [62]	C57BL/6 mice	Corn oil diet or corn oil + fish oil diet for 5 weeks	Omega-6 PUFAs enriched the microbiota with *Enterobacteriaceae* family, omega-3 PUFA enriched microbiota with *Lactobacillus* and *Bifidobacteria* genera of *Firmicutes* phylum.
Mokkala et al. (2016) [69]	Pregnant women	Diet with high intake of omega-3 PUFAs	Pregnant women with high intake of omega-3 PUFAs have shown higher abundance of *F. prausnitzii* species of *Firmicutes* phylum and a lower abundance of *Bacteroides* genera of *Bacteroidetes* phylum.

**Table 3:** Summarized studies investigating the omega-3 effects on microbiota composition in stressed and depressed animal models.

Studies	Studied Population	Diets	Main Outcomes
Robertson et al. (2017) [92]	C57BL/6J mice	Control standard chow or omega-3 PUFA supplemented diet contained 1 g EPA + DHA/100 g diet (O3+), or omega-3 PUFA deficient diet (O3−)	O3+ diet leads to an increase of the abundance of *Bifidobacterium* and *Lactobacillus* genera; enhances cognition and dampens HPA axis activity.
Pusceddu et al. (2015) [99]	Maternally separated female rats	Saline water or EPA/DHA 0.4 g/kg/day (low dose) or EPA/DHA 1 g/kg/day (high dose)	Long-term administration of high dose of EPA/DHA leads to restoration of the normal *Firmicutes/Bacteroidetes* phyla ratio; increases level of the butyrate-producing bacteria *Butyrivibrio* genus; increases the levels of several members of anti-inflammatory *Actinobacteria* phylum (such as *Aerococcus* genus); decreases the abundance of pro-inflammatory *Proteobacteria* phylum (such as *Undibacterium* genus); and decreases other pro-inflammatory bacteria genera including *Akkermansia* and *Flexibacter*.
Davis et al. (2016) [103]	Socially isolated C57BL/6J mice	Control diet (modified AIN-93G diet composed by soybean, soy, and corn oils) or modified AIN-93G diet with the addition of 0.1% by weight DHA or modified AIN-93G diet with the addition of 1% by weight DHA	Addition of DHA leads to sex-specific compositional shifts within the *Firmicutes* phylum, more accentuated in male than in female, with an increase of *Allobaculum* genus (SCFAs-producing bacteria) and a decrease of *Ruminococcus* genus (involved in tryptophan metabolism).
